# The Effect of Dietary Factors on Cancer

**DOI:** 10.3390/ijms24076802

**Published:** 2023-04-06

**Authors:** Monica Benvenuto, Roberto Bei

**Affiliations:** 1 Department of Clinical Sciences and Translational Medicine, University of Rome “Tor Vergata”, 00133 Rome, Italy; monica.benvenuto@unicamillus.org; 2 Departmental Faculty of Medicine and Surgery, Saint Camillus International University of Health and Medical Sciences, 00131 Rome, Italy

The effects of dietary factors on cancer have been widely studied for several decades. The American Cancer Society estimates that in 2023, approximately 1,958,310 new cases of cancer will be diagnosed, and 609,820 cancer deaths will occur in the United States [1]. Diet has been identified as a key modifiable risk factor for several types of cancer, including colorectal, breast, prostate, and lung cancer. Indeed, the composition of an individual’s diet can affect nutritional biomarkers (biomarkers of exposure, effect/function, and health/disease and physiological status), which can, in turn, affect cancer risk [2,3]. This Special Issue, “The Effect of Dietary Factors on Cancer 2.0”, in the International Journal of Molecular Sciences, comprises eight original research papers and nine review articles. These papers highlight the current research on the anti-tumoral effects of several dietary factors, including their in vitro and in vivo mechanisms of action, in different types of cancers. The nutraceuticals, which are natural components of the diet, including purified substances from foods, plant extracts, dietary supplements, vitamins, and phytonutrients, possess many biological activities (anti-microbial, antioxidant, anti-inflammatory, anti-viral, anti-cancer, and immunomodulatory) that can influence the prevention and/or treatment of diseases, such as cancer [4,5,6,7,8].

The review by Matsushita et al. suggested that, although the relationship between diet, nutrition, and prostate cancer is not well-understood, an intervention targeting dietary patterns may be a potential avenue for prostate cancer prevention. Indeed, gut microbiota and nutrients, such as fat, protein, carbohydrates, vitamins, polyphenols, and also zinc, may affect the development and progression of prostate cancer [9,10]. Similarly, the review by Hayashi et al. pointed out how lifestyle and a pro-inflammatory diet could be risk factors for bladder cancer because of the induction of chronic systemic inflammation. On the other hand, fruit and vegetable intake play a protective role. Thus, the diet has the potential to modulate the carcinogenesis process [11]. It has been also reported that polyphenols present in foods and beverages of plant origin can efficiently modulate autophagy in several types of cancer. The role of autophagy in cancer is debated, as autophagy can both induce and suppress tumour growth. Accordingly, the modulation of autophagy by polyphenols could represent a suitable tool for developing novel therapies to fight cancer [12].

In their original research paper, Colapietro et al. elucidated, for the first time, the in vitro and in vivo anti-cancer properties of crocetin on glioblastoma. Crocetin, a saffron extract, was demonstrated to possess anti-proliferative and pro-differentiative effects that are able to activate apoptosis and reduce cell migration and wound repair. Moreover, crocetin was able to counteract the growth of glioma cells in vivo in subcutaneous xenograft murine and orthotopic intra-brain tumour models [13]. Khan et al. demonstrated the role of the well-known polyphenol curcumin in papillary thyroid cancer (PTC). Curcumin had cytotoxic effects in PTC cell lines by stimulating apoptosis, inhibiting the constitutively active Janus Kinase/Signal Transducer and the Activator of the Transcription 3 (JAK/STAT3) signalling pathway and enhancing the anti-tumoral activity of cisplatin. These findings suggest that the use of curcumin in combination with chemotherapeutic agents could improve the clinical outcomes of PTC patients [14]. Indeed, preclinical and clinical studies have shown how dietary and medicinal plant polyphenols can overcome multidrug resistance to chemotherapeutic agents in breast, lung, colorectal, and prostate cancer by modulating different signalling pathways [15].

Two research articles demonstrate the inhibition of migratory properties of prostate cancer cells and triple-negative breast cancer cells by the grape-derived phytochemical resveratrol and by soybean phytoalexins glyceollins [16,17]. The first study shows how resveratrol in vitro inhibits the stroma–epithelium interaction mediated by the hepatocyte growth factor (HGF) and is thus able to control the epithelial-to-mesenchymal transition (EMT) and inhibit prostate cancer cells migration [16]. The second research paper demonstrates that glyceollins are novel aryl hydrocarbon receptor (AhR) ligands and show anti-migration activity in the MDA-MB-231 mammary cancer cells by decreasing N-cadherin gene expression and modulating the expression of Chemokine (C-C motif) ligand 2 (CCL2) and PDZ-LIM domain protein 4 (PDLIM4) genes [17]. In addition, Itkin et al. explore the role of flavonoids as natural AhR ligands in renal cancer, suggesting that aminoflavone (AFP 464) and benzothiazole (5F 203) have potential therapeutic effects in this type of cancer [18].

Different studies have reported the anti-cancer effects of dietary factors in colorectal cancer (CRC). The chemo-preventive potential of a combined treatment of microencapsulated probiotics (*Bifidobacterium longum* BAA-999, BF) and lycopene (LYC) in an azoxymethane (AOM)/dextran sulphate sodium (DSS)-induced CRC model has been evaluated. The combined treatment modulated the major determinant of CRC pathogenesis, the insulin-like growth factor-I/Insulin-like growth factor-I receptor (IGF-1/IGF-1R) system [19]. The study by Gao et al. suggests a novel mechanism of action in tumour suppression by 3,3′-Diindolylmethane (DIM), a naturally derived chemo-preventive compound formed in the acidic environment of the stomach after the ingestion of glucobrassicin: an indole glucosinolate enriched in cruciferous vegetables. DIM directly inhibits an oncogenic mouse double minute 2 homologue (MDM2) at mRNA and protein levels, inhibits cancer cell proliferation, and induces cell cycle arrest and apoptosis in CRC cells. Moreover, DIM showed synergistic anti-cancer effects when applied in combination with cis-imidazoline MDM2 inhibitors, thus providing a novel potential therapeutic strategy for CRC [20]. Hwang et al. demonstrated the anti-cancer potential of the oral administration of zerumbone, the main component of the subtropical ginger plant *Zingiber zerumbet*, which was able to reduce colonic inflammation, hyperplasia, colonic polyp number, and to prevent macroadenoma progression [21]. In the same in vivo model, high salt diet (HSD) consumption was also demonstrated to decrease enterotoxigenic *Bacteroides fragilis* (ETBF)-colitis and carcinogenesis in ETBF-colonized mice with AOM/DSS-induced tumorigenesis by affecting the gut microbiome and immune system. Thus, they suggested how an HSD, although not recommended, could be beneficial under certain conditions [22]. Indeed, as pointed out in the review by Bojková et al., a high fat diet (HFD) traditionally has been considered unhealthy. However, the harmful impacts of HFD on human health mainly stem from the excessive accumulation of adipose tissue, particularly visceral adipose tissue, and the resulting induction of inflammation. Human studies have indicated that a reduction in total fat intake does not significantly impact the risk of developing cancer. Rather, the type of fatty acids in the diet plays a crucial role. Thus, additional studies are needed to fully establish the role of fatty acids in regulating cell proliferation, thus leading to the development of an effective approach for cancer prevention and treatment [23].

Two review papers reporting preclinical and clinical studies demonstrated that the primary compounds found in the *Cannabis sativa* L. plant, known as cannabinoids, reduce tumour growth and trigger apoptosis in melanoma [24] and prostate cancer [25]. Even though the use of cannabis is prohibited in many countries, there has been a renewed interest in exploring the potential health benefits of these plant-derived compounds in recent years.

Collectively, these studies support the role of dietary factors in the prevention and treatment of cancer. However, the effects of dietary factors on cancer are complex and multifaceted. While many dietary components have been shown to possess anti-cancer properties, their effectiveness in vivo can be limited by their poor bioavailability. The effectiveness of these components could be increased through the application of novel techniques, such as nanotechnology, to develop novel formulations with increased bioavailability, solubility, and stability in the human body, such as nanosuspensions, solid lipid nanoparticles, liposomes, gold nanoparticles, polymeric nanoparticles. Epidemiological studies have also identified the role of nutrition in cancer risk and survival, and the development of functional foods enriched with anti-cancer compounds can provide a convenient and effective modality for individuals to improve their diet and reduce their risk of cancer. Further studies are needed to completely understand the mechanisms of action of dietary factors on cancer cells and to develop effective strategies for cancer prevention and treatment.
